# The transmission of trauma inside survivor families: from Shoah to current conflicts and persecutions

**DOI:** 10.3402/ejpt.v4i0.22985

**Published:** 2013-12-05

## XIII ESTSS Conference: “Trauma and its clinical pathways: PTSD and beyond”, Bologna, June 2013 ORAL, JUNE 8 HALL SYDNEY

## Open Papers: Contesti traumatici complessi

### La trasmissione del trauma nelle famiglie dei sopravvissuti: dalla Shoah ai più recenti conflitti e persecuzioni 15.45–16.00

#### Valentina Cassese “Carlo Bo” Dipartimento di Scienze dell'Uomo via Saffi, Università degli Studi di Urbino, Urbino, Italy

Le teorie sulla transgenerazionalità del trauma offrono modelli esplicativi della sofferenza delle seconde e terze generazioni dei superstiti agli orrori di conflitti e persecuzioni cercando quindi di capire i meccanismi per cui individui nati anche in tempi e luoghi lontani dagli eventi sperimentano gli effetti come se avessero vissuto l'esperienza direttamente. Questo lavoro sintetizza il dibattito relativo ai meccanismi che permettono la trasmissione tra generazioni ed alle difficoltà psichiche registrate in ciascuna di esse. Infatti sebbene solo un numero relativamente piccolo di sopravvissuti alle violenze di massa presenti malattie mentali tali da richiedere un intervento psichiatrico intenso, la maggior parte di loro soffre di problemi mentali di minore gravità ma di lunga durata e queste sofferenze sembrano perpetrarsi in qualche modo sui loro discendenti. In particolare sarà esaminato il contributo della dr.ssa Wardi che ha osservato e proposto la necessità di un sostegno terapeutico alle generazioni successive ai sopravvissuti dato che esiste un profondo legame fra l'impossibilità di attuare il naturale processo di individuazione rispetto alla famiglia d'origine e il ruolo che questi individui hanno all'interno della famiglia stessa. La seconda generazione avrebbe il compito di colmare l'immenso vuoto lasciato nelle vite dei propri genitori, rappresentando per questi ultimi un anello di congiunzione fra passato e presente, la *candela della memoria* accesa affinchè ne rimanga il ricordo.

**References**

Wardi D. (1992), *Le candele della memoria: i figli dei sopravvissuti all'Olocausto*, Sansoni (1999).

Zajde N. (1993), *Enfant de survivant. La transmission du trumatisme chez les enfantes Juifs survivantes de l'extermination nazie*, Edition Odile Jacob, Paris.

Nicolò Corigliano A. M. (1996), Il transgenerazionale tra mito e segreto. *Interazioni*, 1.

### The transmission of trauma inside survivor families: from Shoah to current conflicts and persecutions

Theories of trauma transmission offer explanatory models of the suffering of the second and third generations of the survivors of conflict and persecution horrors then trying to understand the mechanisms by which individuals born also in times and places far away from those traumatic events experience the effects *as if* they had lived through the experience directly. This work summarizes the debate concerning the mechanisms that allow the transmission between the generations and psychological difficulties registered in each of them. Indeed, only a relatively small number of survivors of mass violence were present with mental illness, such as to require intense psychiatric intervention, most of them suffer from mental problems of lesser gravity but long term and these sufferings seem to be perpetrated in some way on their descendants. Particularly, by examining the contribution of Dr. Wardi, it has been observed and proposed that the need for therapeutic support for survivors’ subsequent generations, there is a deep link between this status and the inability to implement the natural process of identification. The second generation seems to have the task of filling the huge vacuum left in the lives of their parents, representing for the latter a ring of conjunction between past and present, a *candle of memory* lit, so that the remembrance can remain.

